# Synergistic preservation of fresh cut fruits and vegetables through cold chain logistics and antagonistic microorganisms: mechanisms and emerging applications

**DOI:** 10.3389/fmicb.2026.1925132

**Published:** 2026-09-22

**Authors:** Jiaying Duan, Lili Sun, Min Zhang, Peiyi Tian, Qinyang Cheng, Jing Sun, Bangdi Liu, Muhammad Tufail, Maha Abdullah Alwaili, Xuexia Guo, Zhigang Li

**Affiliations:** 1 College of Food Science and Engineering, Shanxi Agricultural University, Taigu, Shanxi, China; 2 Dongying Agricultural Comprehensive Service Center, Dongying, Shandong, China; 3 Academy of Agricultural Planning and Engineering, Ministry of Agriculture and Rural Affairs, Beijing, China; 4 Key Laboratory of Agro-Products Primary Processing, Ministry of Agriculture and Rural Affairs of China, Beijing, China; 5 College of Life Science and Food Engineering, Hebei University of Engineering, Handan, China; 6 School of Economics, Beijing Technology and Business University, Beijing, China; 7 Department of Biology, College of Science, Princess Nourah bint Abdulrahman University, Riyadh, Saudi Arabia

**Keywords:** antibacterial mechanism, biological antagonists, cold chain logistics, fresh-cut fruits and vegetables, synergistic preservation

## Abstract

Fresh-cut fruits and vegetables are widely popular owing to their pleasant texture, rich nutrients and convenient consumption. Nevertheless, microbial contamination and physiological deterioration frequently emerge during processing and cold chain distribution, triggering quality degradation and shortened shelf life. Conventional single cold storage fails to efficiently suppress psychrotrophic spoilage microorganisms, whereas chemical preservatives carry hidden risks of toxic residues. Current research on antagonistic microorganisms remains fragmented, with inadequate systematic collation of synergistic relationships among low-temperature conditions, antibacterial performance, fruit quality and strain biosafety; this deficiency hinders industrial popularization. This review thus aims to fill these research gaps. Authoritative Chinese and English literature was retrieved and screened under unified inclusion and exclusion criteria to elaborate the interactions between cold chain systems and antagonistic microbes. Low temperature stabilizes the metabolic traits of antagonistic strains, sustains persistent antagonistic activity and reduces biosafety risks. Standardized cold chain management maintains flesh firmness and bioactive ingredients and inhibits the accumulation of spoilage metabolites. This review also summarizes advanced technologies including precise strain screening, sustained-release carrier fabrication and AI-assisted optimization. Collectively, this paper clarifies the synergistic mechanisms linking cold chain and antagonistic microorganisms, addresses the lack of systematization in existing studies, provides theoretical support for green preservation in commercial fresh-cut cold chain logistics, and guides future research targeting cold-adapted antagonistic strains.

## 1 Introduction

### 1.1 Research background and significance

In recent years, fresh-cut fruits and vegetables have gained growing favor among consumers, driven by rising living standards, increasing health consciousness, and an accelerating pace of life. Fresh-cut fruits and vegetables refer to products that undergo a series of processing steps-such as grading, trimming, selecting, washing, cutting, preservation, and packaging to maintain their fresh state. They are characterized by freshness, nutrition, and convenience (Wu and Lou, 2001).

Fresh-cut fruits and vegetables originated in the United States in the 1950s, underwent commercialization in the 1960s, and witnessed rapid development in Europe and Japan during the 1980s (Cheng et al., 2019). Fresh-cut fruits and vegetables account for 25% of the total fruit and vegetable sales in the United States, and this share continues to grow. By 2024, the fresh vegetable market size is valued at USD 949.8 billion (Research and Markets, 2025). In Italy, packaged fresh-cut vegetables are very popular, and in 2024, the market size was approximately 848 million euros (Yang, 2024). In Japan, supermarkets are the main sales channel for fresh-cut fruits. The consumption of processed vegetables increases every year, and prepared vegetables are growing the fastest (Li, 2019). In China, fresh-cut fruits and vegetables started in the 1990s. In the past 10 years, the markets have grown rapidly (Analysis and Investment Trend Forecast Report, 2025; Beijing Boyan Zhishang Information Consulting Co., Ltd., 2025; Market Prospect Forecast and Investment Value Evaluation Analysis Report, 2025). In 2024, the fresh-cut vegetable market size was over 3.5 trillion yuan, and online channels showed significant growth (The Paper Paike, 2025). Eastern China is the main consumption market, and younger consumers and families are the main buyers (Mujin, 2023; Beijing Boyan Media Information Consulting Co., Ltd., 2025).

Fresh-cut fruits and vegetables are popular and important in the produce market. National demand is increasing, and they are becoming a primary way people consume fruits and vegetables (Wei et al., 2026; Hu et al., 2023). But these products also have rotting problems. This spoilage is caused by two main factors: external microbial contamination and internal metabolic problems. Fresh-cut fruits and vegetables are more prone to harmful bacteria during storage and transport because the outer skin is removed. The nutrients on the cut surfaces provide good conditions for spoilage microorganisms to grow and reproduce, resulting in rapid growth. This affects the safety and also hurts the look and taste of the products (Wang et al., 2025a). In addition, most fruits and vegetables produce the plant hormone ethylene. If they are cut or bruised, they quickly produce a large amount of ethylene and respire faster, and they also produce more secondary substances, such as polyphenols and flavonoids (Shi et al., 2023). Also, because mechanical tissue damage, enzymes like PPO and POD come into contact with phenolic compounds, and they turn them into melanin, and thus this causes browning (Wang et al., 2025b).

To solve these problems, many countries use technologies that combine cold storage and real-time monitoring. This helps reduce the breathing rate of fresh-cut fruits and vegetables and also limits spoilage microorganisms on the surface. Developed countries, such as those in Europe and America, use their good cold chain knowledge and systems. In recent years, they have paid more attention to cold chain sustainability and the use of technologies such as RFID and IoT to improve work efficiency (Mustafa et al., 2024). Related research includes smart tracking systems, real-time monitoring solutions, analysis of the 3E (Efficiency, Economy, Environment) features of cold storage, and machine learning-based prediction methods for energy and environment management (Luo et al., 2016; Gillespie et al., 2023; Zarabadi and Mafi, 2024; Alkhulaifi et al., 2024).

China has also attached great importance to the construction and upgrading of cold chain infrastructure. To solve important industry problems, during the 14th Five-Year Plan, ministries such as the Ministry of Agriculture and Rural Affairs and the National Development and Reform Commission worked together to build cold storage and preservation facilities for farm products. This plan aims to build a full cold chain from production to sales, including promoting mobile cold storage and pre-cooling, upgrading refrigerated transport, and using recyclable packaging and retail fridges to keep fruits and vegetables fresh and reduce losses (General Office of the State Council, 2021). This strategy inhibits spoilage microorganisms at low temperatures, reduces the respiration rate, and delays surface browning of fresh-cut produce.

However, while low temperatures can inhibit the growth of most microorganisms, they cannot effectively suppress psychrotrophic bacteria on the surface of freshly cut fruits and vegetables, such as *Pseudomonas* spp., *Clostridium botulinum*, and *Listeria monocytogenes*, and the proliferation of these microorganisms remains a key factor in causing spoilage (Li et al., 2026; Chen et al., 2024; Chen et al., 2012). Researchers have used cold storage and chemical preservatives, such as potassium sorbate, ascorbic acid, and citric acid, to solve these problems. This approach is widely adopted for its low cost, straightforward operation and diverse available formulations. However, it can leave chemicals, change taste, and pose safety risks. Some preservatives, such as chlorine, sodium hypochlorite, and chlorine dioxide, can cause carcinogens. Sulfites can cause allergies in patients with asthma and have adverse effects on health. So developed countries, such as the US, have started to limit their use in fresh-cut products (Luo et al., 2010). And fresh-cut fruits and vegetables can be kept fresh by changing the gases around them or by changing the surface environment. D’Aquino et al. (2016) found that micro-perforated films can keep CO₂ at 2–4 kPa and O₂ at 15–18 kPa and keep humidity near 100%. This can lower respiration, slow the loss of bioactive compounds, and keep cherries flesh firmness The experiment by Meyer et al. (2024) demonstrated that bacteria belonging to the genera *Pseudomonas* spp. and *Bacillus* spp. can inhibit mycelial growth and reduce the presence of *Sclerotinia sclerotium*, so this decreases its associated postharvest diseases in carrots. This not only meets the current consumer demand for efficient, green, and healthy products but also achieves better preservation effects.

In summary, the limitations of single cold storage and chemical preservatives create urgent industrial demands for cold-chain biopreservation. Relevant existing studies are scattered, lacking systematic integration of low-temperature response, antibacterial mechanism, fruit quality and biosafety, which demonstrates the necessity of this review. Distinct from previous reviews that separately discuss cold storage or antagonistic microbes, this work centers on cold chain scenarios, integrates multi-dimensional interaction rules, and categorizes cold-adapted optimization technologies in a targeted manner. Such targeted collation endows this review with unique reference value for follow-up basic research and industrial application.

### 1.2 Literature search and screening methodology

To ensure the comprehensiveness and reliability of this review, standardized literature retrieval and screening procedures were implemented. Retrieval was performed across three international databases, including Web of Science Core Collection, Scopus, and ScienceDirect, as well as two mainstream Chinese databases (China National Knowledge Infrastructure and Wanfang Data). Boolean operators were used to construct the search strings: “fresh-cut fruits and vegetables” AND (“cold chain logistics” OR “low-temperature storage”) AND (“antagonistic microorganisms” OR “biocontrol agents” OR “biological preservation agents”), and equivalent Chinese search terms were adopted for the Chinese databases. The primary retrieval timeframe covered publications from January 2000 to March 2026. In addition, several landmark classic papers published before 2000 were manually traced and supplemented based on citation relationships in core reviews, to establish the historical research background of this field.

Literature screening followed unified inclusion and exclusion criteria: eligible papers focused on fresh-cut fruits and vegetables, involved low-temperature or cold storage conditions, reported applications of antagonistic microorganisms for preservation, and contained complete quantitative experimental data; by contrast, studies on intact horticultural products, single preservation technologies without synergistic analysis, duplicate publications, conference abstracts, and incomplete patents were excluded. A total of 1,286 preliminary records were retrieved, and 972 non-duplicate articles remained after deduplication. After preliminary screening by titles and abstracts, 214 potentially relevant documents were retained. Finally, 152 core references (including a small number of pre-2000 classic papers) with high research value were confirmed via full-text reading and quality evaluation, for subsequent categorization and discussion in this manuscript.

### 1.3 Current research status (at home and abroad)

In recent years, researchers both domestically and internationally have focused on exploring integrated approaches that combine low-temperature storage with other preservation technologies. By leveraging the synergistic effects of physical, chemical, and biological methods, they aim to further extend the shelf life of fresh-cut fruits and vegetables. These studies have offered new insights and directions for the innovation and development of preservation techniques in this field.

#### 1.3.1 Combination of low temperature and chemical preservation methods

Chemical preservation methods primarily involve the direct application of antimicrobial agent to the surface of fresh-cut fruits and vegetables, with the objectives of inhibiting microbial growth, delaying enzymatic browning, and regulating physiological metabolism. Zhuang et al. (2025) investigated the application of an active packaging film incorporated with the natural antioxidant AOB for preserving fresh cherries under 4 °C cold storage. The treated cherries exhibited minimal firmness loss, the most stable color, and a significantly reduced decay rate. Furthermore, this approach effectively slowed the increase in anthocyanin content and maintained better nutritional quality and flavor. Akbas and Lm ez (2007) treated fresh-cut iceberg lettuce with lactic acid and citric acid, finding that the counts of both psychotropic and mesophilic bacteria decreased after treatment, effectively extending the product’s shelf life. Furthermore, studies have shown that postharvest application of 1-methylcyclopropene (1-MCP) to papaya fruit stored at 4 °C not only enhances the fruit’s chilling tolerance but also better maintains its color and firmness (Wang, 2025) Bai Wuriliga (Bai et al., 2025) immersed fresh-cut *Tricholoma mongolicum* mushrooms in slightly acidic electrolyzed water (SAEW) and stored them at 4 °C. The results indicated that the SAEW-treated fresh-cut mushrooms exhibited strong scavenging capabilities against DPPH, hydroxyl, and superoxide anion radicals. These results indicate that SAEW exhibits a favorable preservation effect on fresh-cut mushrooms.

#### 1.3.2 Combination of low-temperature and other physical preservation methods

The combination of low-temperature and physical methods aims to significantly enhance preservation efficacy by regulating the storage microenvironment and physiological state of Fresh-cut fruits and vegetables, while avoiding the introduction of exogenous chemicals. Yuan et al. (2024b) experimentally confirmed that vacuum packaging (VP) treatment can suppress the cutting-induced activities of phenylalanine ammonia-lyase (PAL) and polyphenol oxidase (PPO), thereby inhibiting the biosynthesis and oxidative polymerization of phenolic compounds. Combined with low-temperature storage, this method effectively inhibits browning in fresh-cut taro, reduces the total count of pathogenic microorganisms, and maintains its storage quality, representing a simple and practical physical preservation technology. Cao et al. (2010) found that strawberries with ultrasound (40 kHz, 350 W, 10 min) and stored at 5 °C and 80–90% humidity for 8 days showed a drop in decay rate from 23 to 13%. The total bacterial count decreased from (3.23 ± 0.04) log CFU/g to (2.43 ± 0.02) log CFU/g, and the mold and yeast numbers decreased from (3.88 ± 0.07) log CFU/g to (3.02 ± 0.02) log CFU/g, which strongly slowed postharvest decay during storage.

#### 1.3.3 Combination of low-temperature and biological preservation methods

Consumer demand for food safety and green consumption is going up, and so low-temperature storage plus biological preservatives from plants and animals has become a hot research topic. For example, Wang et al. (2004) used L-cysteine and low-temperature modified atmosphere storage together, which delayed aging and skin browning of longan, improving its quality and market value. Similarly, Zou et al. (2025) put galangal oil and curcumin into a sodium alginate film to keep fresh-cut pineapple fresh; under refrigeration, this film slowly releases antimicrobial components, and so it stops spoilage from microbes. Also, Rojas-Graü et al. (2007) used an apple-alginate coating with lemongrass, oregano oil, and vanillin on fresh-cut Fuji apples, and the results showed that these three coatings clearly stopped the growth of bacteria, yeasts, and molds.

#### 1.3.4 Combination of low-temperature and microbial (antagonistic) preservation methods

The integration of low-temperature and antagonistic microbial methods represents an active exploration based on the biocontrol strategy of “using bacteria to combat bacteria”. Du et al. (2025) demonstrated that certain lactic acid bacteria (LAB) maintain robust growth and adaptability under low-temperature conditions. The strains *P. acidilactici* L1 and *L. plantarum* HG1-1 enhance superoxide dismutase (SOD) activity to maintain intracellular reactive oxygen species (ROS) homeostasis and scavenge superoxide anion radicals produced by aerobic metabolism. This mechanism protects bacterial cells from damage and helps them to adapt to low temperatures. The selected LAB strains can stop many spoilage microbes, including psychrotrophic bacteria, and this shows that they could be used for fruit and vegetable preservation. Cao et al. (2023) showed that under cold chain conditions, Nisin can insert into the cell membranes of Gram-positive bacteria more stably and form pores, and this causes cell contents to leak out, and so it stops pathogens like *Listeria monocytogenes* (Charest et al., 2024) and keeps cherry tomatoes fresh. Hosseini et al. (2024) got a combined effect by using low-temperature storage at (0 ± 1) °C and adding *Bacillus subtilis* as a biocontrol agent. This method uses low temperatures to slow fruit metabolism and microbes to control pathogens, which greatly reduces decay from *Botrytis cinerea* and keeps sweet cherries fresh during storage.

## 2 Research progress on different types of antagonistic microorganisms for fresh-cut fruit and vegetable preservation

In the preservation of fresh-cut fruits and vegetables, employing specific antagonistic microorganisms to inhibit spoilage microbes represents a promising green biocontrol strategy. Currently, the antagonistic microorganisms prevalent in both research and application primarily include specific strains of yeasts, *Bacillus* spp., as well as lactic acid bacteria (LAB) and actinomycetes (Qiu et al., 2009). Antagonistic microorganisms suppress pathogens in fresh-cut fruits and vegetables through multiple synergistic mechanisms. Their primary modes of action include production of antimicrobial metabolites; competition for space and nutrients at wound sites; induction of host systemic resistance; mycoparasitism; and enhancement of inhibitory efficacy through synergy with other microorganisms or environmental factors (Li et al., 2012).

### 2.1 Preservation of fresh-cut fruits and vegetables by antagonistic yeasts

Antagonistic yeasts are primarily isolated from the surfaces of healthy fruits and vegetables, orchard soil, as well as leaves and fruits. Commonly studied yeast species include *Meyerozyma guilliermondii*, C*ryptococcus laurentii*, *Candid-a* spp., *Rhodotorula* spp., and *Hanseniaspora uvarum*. These species have been applied to fresh-cut fruits and vegetables such as apples, cherries, grapes, pears, peaches, and tomatoes (Geng et al., 2010). It is generally accepted that the mechanisms by which antagonistic yeasts control postharvest diseases in fruits and vegetables primarily include competition for nutrients and space, direct mycoparasitism of pathogens by yeasts, and the induction of host resistance (Ezzouggari et al., 2024).

Specifically, competition for space and nutrients is considered the primary mechanism of action for the biocontrol effect exerted by yeasts. For example, Tian et al. (2002) demonstrated that when the concentration of the yeast cell suspension increased from 0 to 5 × 10^8^ CFU mL^−1^, both the germination rate and mycelial length of the pathogen decreased. A significant negative correlation was observed between the suspension concentrations of *C. quillirmondii* and *P. membranefaciens* and both the germination rate (*R*^2^ = 0.9064 and 0.9029, respectively) and mycelial length (*R*^2^ = 0.9603 and 0.9642, respectively) of *R. stolonifer,* thus demonstrating nutrient competition between the antagonistic yeasts and the pathogen.

On this basis, antagonistic yeasts can also function by directly parasitizing pathogenic fungi. Cao and Liu (2021) reported that after treatment with the extracellular metabolites of strains FWB1J and YMB1J, the chitinase activity in the treatment groups of the two dominant antagonistic endophytic strains reached its maximum at 36 h, increasing from approximately 4 μg/g to 10 μg/g and from 6 μg/g to 12 μg/g, respectively, indicating that chitinase hydrolyzed chitin to produce large amounts of N-acetylglucosamine, thereby disrupting the integrity of the pathogen cell wall. This result confirms that antagonistic yeasts directly parasitize pathogenic fungi by producing extracellular hydrolytic enzymes, thereby degrading their cell walls and thus exerting a biocontrol effect.

Furthermore, the biocontrol effect of yeasts is also closely related to their influence on host resistance. Droby et al. (2002) studied the antagonistic effect of *Candida* on postharvest *Penicillium* infection in grapes and found that applying a suspension of antagonistic yeast cells to the peel tissue significantly induced the activity of phenylalanine ammonia-lyase (PAL), which increased from approximately 5 μmol·h^−1^·g^−1^ to about 20 μmol·h^−1^·g^−1^ within 48 h after treatment, and promoted the accumulation of phytoalexins. In addition, yeast treatment also induced the accumulation of chitinase (24 kD) and β-1,3-endoglucanase (39 kD) proteins, with the induction of chitinase being particularly pronounced (Figure 1 and Table 1).

**Figure 1:**
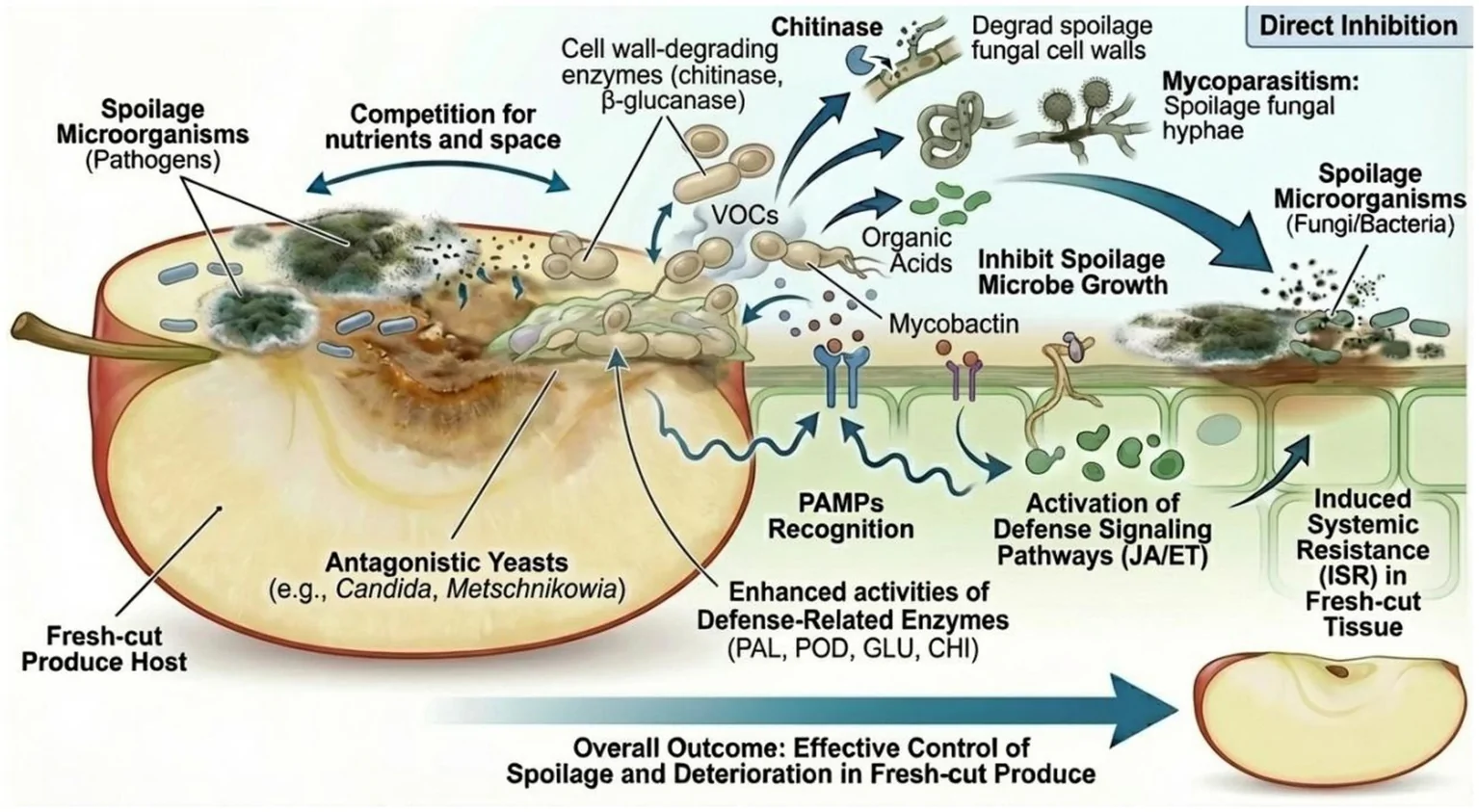
Mechanism of action of antagonistic yeasts.

**Table 1:** Representative antagonistic yeast strains with effective biocontrol efficacy and their modes of action.

Source	Types	Disease name	Types of fruits and vegetables	Mechanism of action	References
Surfaces of healthy fruits, oilfield soil, wild kiwi fruit skins, etc.	*Meyerozyma guilliermondii*	*Botrytis cinerea*	Strawberry	Yeast suspension application enhances strawberry fruit firmness and disease resistance by competing with pathogens for nutrients/space and inducing defense responses (elevated POD/PPO activity, cell wall hardening).	Ding et al. (2010)
		Anthracnose	Loquat	*Meyerozyma guilliermondii*Y35-1 controls loquat postharvest decay by directly adhering to and parasitizing anthracnose pathogen hyphae, inhibiting their growth.	Zhao et al. (2019)
Natural environments such as soil and rice, as well as the peel of healthy fruits	*Cryptococcus laurentii*	Citrus sour rot	Citrus	*Cryptococcus laurentii* controls citrus sour rot through nutrient/space competition and induction of host defense enzymes (e.g., PPO, POD).	Liu et al. (2010)
		Gray mold	Strawberry	Suppression of strawberry gray mold by *Cryptococcus laurentii* through rapid nutrient utilization and site competition with Pathogen.	Zheng et al. (2003)
		Gray mold, Rhizopus rot	Cherry tomato	*Cryptococcus laurentii* suppresses pathogen growth on cherry tomatoes through nutrient and site competition, thereby indirectly inducing fruit disease resistance.	Lai (2019)
		Blue mold	Grapefruit	Enhanced systemic resistance to blue mold in grapefruit by *Cryptococcus laurentii* induced with carboxymethyl chitosan, via activation of phenylpropanoid metabolism and boosted defense enzymes/compounds.	Tian et al. (2023)
		Rhizopus rot	Strawberry	Cryptococcus laurentii suppresses Rhizopus rot in strawberry wounds by rapid colonization and competition with the pathogen for nutrients and space.	Zhang et al. (2007)
Surfaces of healthy fruits and vegetables, leaves, soil, and other natural environments	*Candida oleophila*	Blue mold	Apple	*Candida oleophila* suppresses blue mold in apple wounds by rapid colonization to compete for nutrients/space and by inducing enhanced activity of defense-related enzymes (POD, PPO, PAL).	Cai et al. (2018)
		Banana crown rot	Banana	*Candida oleophila* inhibits banana crown rot by rapidly utilizing nutrients to outcompete the pathogen and preemptively colonizing crown wounds to occupy space.	Lassois et al. (2008)
		Tuber callus	Potato	*Candida oleophila* reduces disease severity in potato tubers by rapidly colonizing wounds, consuming nutrients, and occupying space to inhibit *Fusarium sulphureum* infection.	Zheng (2020)
Surfaces of healthy produce and orchard soil in natural environments	*Rhodotorula*	Blue mold Gray mold	Pear	The yeast *Rhodotorula mucilaginosa* controls postharvest decay in pear fruits by rapidly colonizing wounds through nutrient and space competition, forming a protective barrier that prevents the invasion of blue mold and gray mold pathogens.	Shu et al. (2015)
		Blue mold	Citrus	The yeast *Rhodotorula glutinis* suppresses pathogen growth through nutrient and space competition and by directly attaching to and parasitizing pathogen hyphae.	Sun et al. (2003)
From healthy produce surfaces and orchard soil.	*Debaryomyces hansenii*	Soft rot	Strawberry	*Debaryomyces hansenii* controls soft rot in strawberry wounds through preemptive colonization and nutrient/space competition, inhibiting the growth of the pathogen.	Zhao L. N. et al. (2024) and Zhao Z. X. et al. (2024)
		Blue mold	Citrus	*Debaryomyces hansenii* directly controls disease in citrus wounds by rapid colonization and preemptive resource occupation through nutrient and space competition, thereby inhibiting *Penicillium* spp. growth.	Yan et al. (2013)

### 2.2 Preservation of fresh-cut fruits and vegetables by antagonistic *Bacillus*

*Bacillus* is widely distributed in nature as a dominant microbial population in soil and various environments and can be isolated from diverse habitats including soil and marine settings (e.g., mangrove areas and marine sediments). Commonly studied strains, such as *Bacillus velezensis*, *B. amyloliquefaciens*, *B. subtilis*, and *B. licheniformis*, have been applied to fresh-cut fruits and vegetables like peaches, grapes, tomatoes, apples, and navel oranges (Liu, 2010). Currently, the mechanisms by which Bacillus controls postharvest diseases mainly involve direct inhibition via secretion of antimicrobial substances, competition for nutrients and space, mycoparasitism, and induction of host resistance (Fan et al., 2018).

Specifically, the secretion of antimicrobial substances to directly inhibit pathogen growth is one of the important biocontrol mechanisms of *Bacillus*. Li et al. (2025) demonstrated that *Bacillus velezensis* B1-4, which formed a transparent halo on specific media, could secrete cellulase and protease, produce siderophores, and fix nitrogen. This strain inhibited *Botrytis cinerea* by 89.13% with an 11.08 mm inhibition zone, directly evidencing the mechanism of pathogen suppression through antimicrobial secretion.

In terms of competition for nutrients and space, *Bacillus* also exhibits significant colonization advantages. Xu (2017) found that *Bacillus amyloliquefaciens* NCPSJ7 rapidly colonized navel orange wounds, with its population increasing from 6.1 to 6.35 log CFU/mL in 24 h and peaking at 6.4 log CFU/mL at 48 h. This persistent colonization competitively preempts nutrients and space, providing long-term protection and significantly extending postharvest storage.

In addition, mycoparasitism is also one of the important ways in which *Bacillus* antagonizes pathogenic fungi. Wang et al. (1996) discovered that the antimicrobial protein X98III, produced by *Bacillus subtilis* strain Bs-98, acts against *Botryosphaeria dothidea* (apple ring rot) by dissolving cell walls, deforming hyphae, and inhibiting spore germination, demonstrating a mycoparasitic mechanism.

In addition to direct antagonistic effects, *Bacillus* can also achieve preservative effects by inducing the host plant’s own disease resistance. Qu (2016) mechanistic study revealed that *Bacillus amyloliquefaciens* BP30 inhibits *Botrytis cinerea* by inducing host defense. PAL activity peaked on day 10 in the inoculation group (77.5% higher than control), and the immersion treatment maintained elevated PAL levels (80.1, 72.8, and 27.0% higher on days 10, 19, and 28, respectively), demonstrating that BP30 enhances pear fruit defense by activating enzymes like PAL (Figure 2 and Table 2).

**Figure 2:**
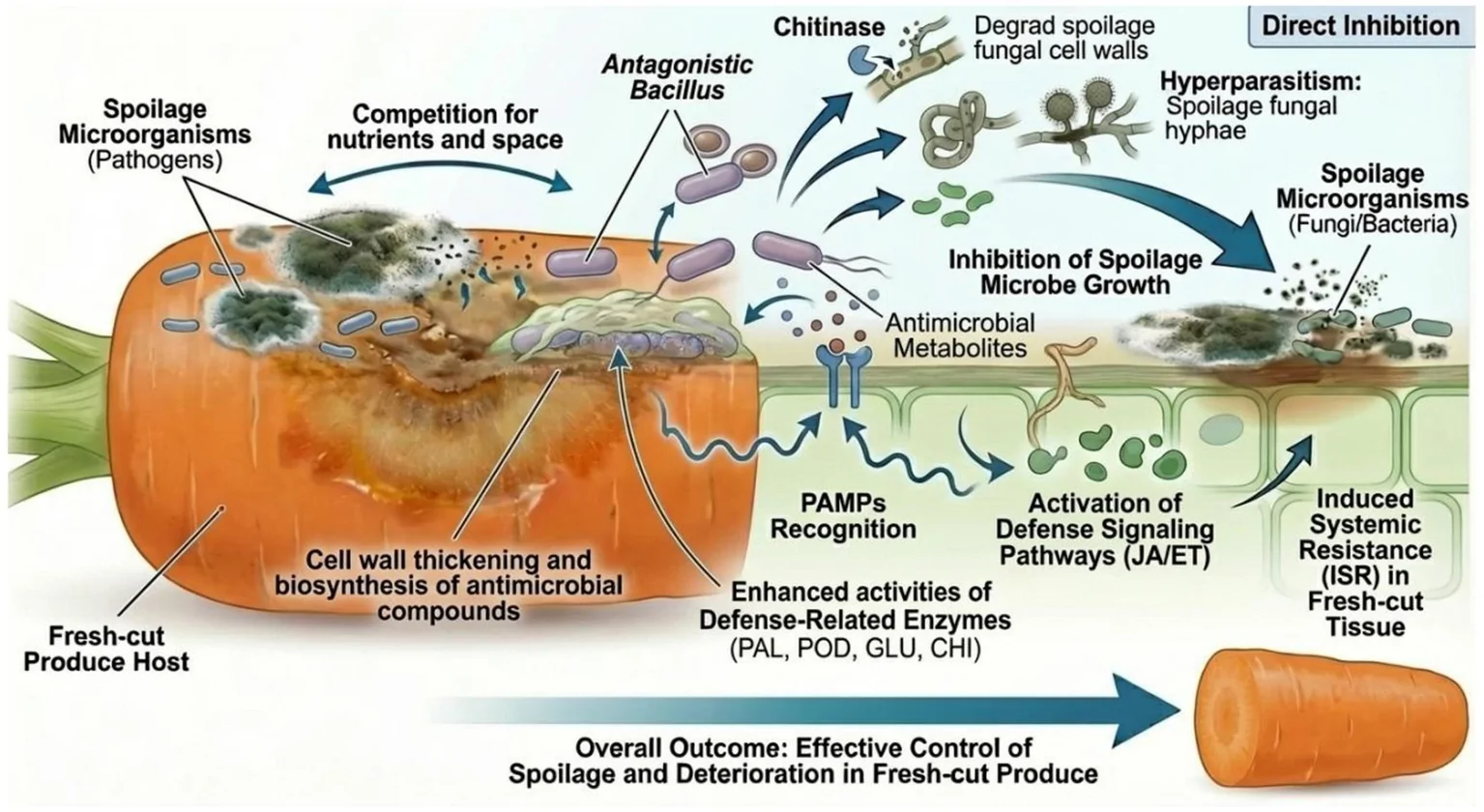
Mechanism of action of antagonistic *Bacillus*.

**Table 2:** Representative *Bacillus* strains with effective biocontrol efficacy and their modes of action.

source	Types	Disease Name	Types of fruits and vegetables	Mechanism of action	References
Rhizospheric soil, leaves, fruit surfaces, rivers, lakes	*Bacillus velezensis*	Brown rot, Soft rot	Peach	*Bacillus velezensis* controls postharvest decay by rapidly colonizing fruit wounds and competing for nutrients, while also secreting antimicrobial substances that directly inhibit pathogens such as brown rot fungi and *Rhizopus* spp.	Zhou H. J. et al. (2025); Zhou M. Y. et al. (2025)
		Potato dry rot	Potato	*Bacillus velezensis* SB023 controls potato dry rot by competing with *Fusarium* spp. for nutrients and space, and by secreting active substances that cause hyphal deformation and metabolic disruption, thereby inhibiting pathogen growth.	Liu et al. (2025)
		Gray mold	Strawberry	*Bacillus velezensis* CY30 effectively controls gray mold on strawberries by secreting antimicrobial substances to inhibit *Botrytis cinerea* growth and by competing for nutrients and space on the fruit surface.	Niu et al. (2025)
		Gray mold	Tomato	*Bacillus velezensis* SM2 achieves biocontrol of tomato gray mold by secreting antimicrobial substances to directly inhibit *Botrytis cinerea* hyphal growth and by inducing the host to enhance protective enzyme activity and accumulate osmoregulatory substances.	Zhang et al. (2024)
From soil, plant roots, leaves to fruit surfaces	*Bacillus amyloliquefaciens*	Apple Alternaria leaf spot	Apple	*Bacillus amyloliquefaciens* wxy-b3 directly inhibits the growth of apple alternaria leaf spot pathogen by secreting lipopeptides that disrupt cell membrane integrity, leading to content leakage and cell death.	Cui et al. (2025)
		Strawberry angular leaf spot	Strawberry	*Bacillus amyloliquefaciens* Y6-7 achieves biocontrol against strawberry angular leaf spot by secreting antimicrobial substances (e.g., lipopeptides and polyketides) to directly inhibit *Xanthomonas fragariae*, and by successfully colonizing strawberry leaves to compete for nutrients and space.	He et al. (2025)
		Cucumber Fusarium wilt	Cucumber	*Bacillus amyloliquefaciens* 9–2 exerts a synergistic biocontrol effect against cucumber Fusarium wilt by secreting antimicrobials that directly disrupt the hyphal morphology of *Fusarium oxysporum f.* sp. *cucumerinum,* and by inducing a significant increase in defense enzyme activity in cucumber leaves.	Fan et al. (2025b)
		Tomato black spot	Tomato	The lipopeptide extract from *Bacillus amyloliquefaciens* effectively inhibits the growth of *Alternaria alternata* by directly disrupting the cellular structures of its hyphae and spores, leading to their distortion, collapse, or rupture.	Fan et al. (2025a)
Rhizosphere soil, leaves, fruit surfaces, air, water bodies, and decaying organic matter.	*Bacillus subtilis*	Anthracnose	Mango	*Bacillus subtilis* 9A directly inhibits the growth and infection of mango anthracnose pathogen (*Colletotrichum* spp.) by secreting extracellular antimicrobial substances that disrupt hyphal membrane permeability, inhibit spore germination, and prevent normal appressorium melanization.	Pan et al. (2013)
		Gray mold	pear	*Bacillus subtilis* BS-3 controls gray mold by preemptive colonization to competitively inhibit the pathogen, and by secreting heat-stable metabolites that directly suppress the hyphal growth and spore germination of *Botrytis cinerea*.	Zhu et al. (2000)
From soil and plant roots to leaves and fruit surfaces.	*Bacillus licheniformis*	Brown rot, Peach twig blight	Peach	Immersion treatment of peach fruits with the culture or antimicrobial proteins of *Bacillus licheniformis* W10 extends shelf life and reduces decay by directly inhibiting pathogen growth through secreted antimicrobial substances.	He and Sun et al. (2020)
		Citrus canker	Citrus	Spraying with a combined suspension of *Bacillus licheniformis* and benzothione effectively controls navel orange canker before disease onset or during the early stages of infection by secreting antimicrobials like chitinase to directly inhibit *Xanthomonas citri*, parasitically disrupt hyphal structures, and compete for nutrients/space.	Yu and Guo (2013)

### 2.3 Preservation of fresh-cut fruits and vegetables by antagonistic actinomycetes

Actinomycetes are widely distributed in terrestrial and aquatic ecosystems, primarily in soil. Common research genera include *Streptomyces* and *Nocardia* (Sharma et al., 2014), which have been applied in the preservation of fruits and vegetables such as strawberries, tomatoes, apples, and grapes. These microorganisms control postharvest diseases primarily through competition for nutrients and space, direct inhibition via the production of antimicrobial metabolites, and induction of host resistance (Jia et al., 2025).

In terms of competition for nutrients and space, Wang (2006) showed that actinomycete SW20 thrives in rhizosphere soil, competing with pathogens for space. While not endophytic, it stably colonizes the cucumber rhizosphere to establish a protective zone.

In addition to competition for nutrients and space, actinomycetes can also directly inhibit pathogens by producing antimicrobial metabolites. The cell-free fermentation filtrate of actinomycete XJ93, studied by Zhao et al. (2018) exhibited strong inhibition against *Botrytis cinerea*, with an inhibition zone of 20.67 mm and a rate of 71.67%, demonstrating a direct inhibitory mechanism.

Furthermore, actinomycetes can also enhance the defense capability of fruits and vegetables by inducing their own resistance. In the experiment by Zhang et al. (2024), treatment with actinomycete agents at 15 and 30 g·plant^−1^ significantly increased jujube yield and fruit quality (with respective increases of 11.46, 12.08, 8.66 and 10.45%, 11.46, 9.15% for single-plant yield, premium fruit weight, and premium fruit rate) and enhanced leaf PPO activity by 41.6 and 45.2% at 30 and 45 g·plant^−1^, indicating an induced resistance mechanism via host defense activation (Figure 3 and Table 3).

**Figure 3:**
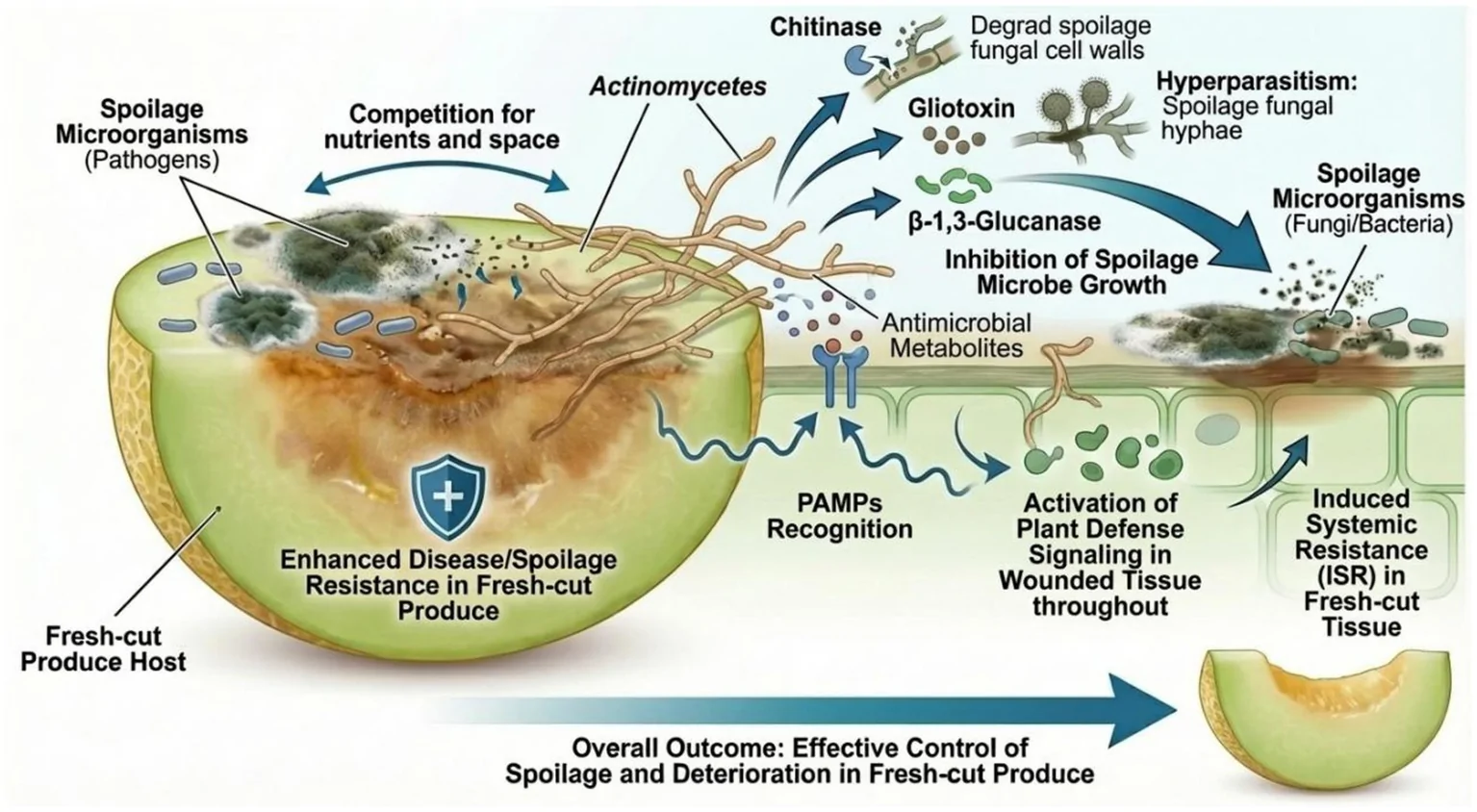
Mechanism of action of antagonistic Actinomycetes.

**Table 3:** Representative actinomycetes with effective biocontrol efficacy and their modes of action.

Source	Types	Disease name	Types of fruits and vegetables	Mechanism of action	References
Natural environments such as soil, water bodies, and decaying organic matter.	*Streptomyces*	Soft rot	Dragon fruit	*Streptomyces* LWL1827 controls pitaya diseases by producing antimicrobial metabolites to directly inhibit pathogens and by inducing the activity of resistance enzymes (PPO, POD, PAL).	Zhang et al. (2023)
		Soft rot	Jujube	*Streptomyces* chemically controls fruit tree diseases by producing antimicrobial metabolites that directly inhibit pathogens, applied through periodic spraying.	Zhao (2010)
		Stem-end rot	Mango	*Streptomyces misionensis* TF78 directly inhibits pathogen growth by producing volatile antimicrobial metabolites, primarily 1,4-dichlorobenzene.	Huang et al. (2021)
		Anthracnose	Banana	*Streptomyces misionensis* TF78 directly inhibits pathogen growth by producing volatile antimicrobial metabolites.	Huang et al. (2021)
		Anthracnose	Strawberry	*Streptomyces* sp. ZZSP-7 directly inhibits the growth of *Colletotrichum* spp., the strawberry anthracnose pathogen, by producing antimicrobial metabolites.	Yao et al. (2021)
		Green mold	Citrus	By producing antimicrobial metabolites (ε-Polylysine), it directly disrupts the cell integrity of *Penicillium*, leading to cellular content leakage.	Lin et al. (2022)
		Gray mold	Grape	Through fumigation with its produced volatile organic compounds (VOCs), it directly inhibits the spore germination and mycelial growth of *Botrytis cinerea*, and disrupts the cellular ultrastructure of the pathogen.	Lu et al. (2024)
		Gray mold	Tomato	By utilizing non-volatile antimicrobial metabolites (e.g., Natamycin) from its fermentation broth, it directly inhibits pathogens.	Lu et al. (2024)
		Bacterial wilt	Tomato	*Streptomyces* sp. NEAU-D18 and NEAU-88 consortia directly inhibit *Ralstonia solanacearum* through nutrient competition and antimicrobial metabolite production, and are applied in controlling tomato bacterial wilt.	Zhang (2021)
In soil, internal healthy plant tissues, etc.	*Nocardia*	Soft rot	Sweet potato	Strain MI-S-24 effectively controls postharvest soft rot of sweet potato, caused by *Rhizopus stolonifer*, through its production of volatile organic compounds (VOCs).	Gomaa et al. (2025)
		Bacterial spot	Tomato	*Nocardia vaccinii* IMV B-7405 controls disease through direct inhibition of plant pathogens by producing antimicrobial metabolites.	Piatetska et al. (2019)
		Bacterial spot	Chili	*Nocardia vaccinii* IMV B-7405 also directly inhibits plant pathogens by producing antimicrobial metabolites.	Piatetska et al. (2019)
		Root rot, Anthracnose	Strawberry	*Nocardioides luteus* CM-13–16 directly inhibits pathogens by producing antimicrobial metabolites while also promoting plant growth through the synthesis of the auxin IAA.	Institute of Agricultural Resources and Regional Planning, Chinese Academy of Agricultural Sciences (2020)

### 2.4 Preservation of fresh-cut fruits and vegetables by antagonistic *Pseudomonas*

Antagonistic *Pseudomonas* are primarily isolated from rhizosphere soil or plant tissues (Cui et al., 2024). Commonly studied species include *Pseudomonas fluorescens*, *P. protegens*, *P. aeruginosa,* and *P. syringae*. They have been applied in preserving fruits and vegetables like apple, tomato, and melon. They control postharvest diseases mainly through nutrient and space competition, production of antimicrobial substances (e.g., antibiotics and siderophores), and induction of host resistance (He, 2025).

Song et al. (2025) demonstrated that colonization by the biocontrol strain P1 significantly reduced the population of *Fusarium oxysporum* in soil. In the LD treatment (pathogen only), the *F. oxysporum* gene copy number exceeded 5.0 log(copies/g dry soil). In contrast, in the LP2 treatment (co-inoculated with P1), it decreased to approximately 3.0 log(copies/g dry soil). This sharp decline confirms that P1 colonization in the rhizosphere greatly limited pathogen survival and reproduction through competition for nutrients and space.

In addition to competition for nutrients and space, the production of antimicrobial substances to directly inhibit pathogens is also an important biocontrol mechanism of *Pseudomonas*. Ma et al. (2025a) found that strains P2-1, P3-1, and P3-4 produce both protease and siderophores. Their study specified that pectinase and protease inhibit *F. oxysporum* by degrading its cell wall to enter the cells. Additionally, the produced siderophores tightly chelate environmental iron, depriving the pathogen of this essential element. After treatment with these strains, the disease indices of watermelon plants were 34.38, 35.94, and 34.38, respectively, all lower than the control (CK). The corresponding control efficacies against watermelon Fusarium wilt were 63.93, 62.29, and 63.93%, demonstrating a direct inhibition mechanism via antimicrobial production.

In addition to the direct antagonistic effects mentioned above, *Pseudomonas* can also enhance its defense capability by inducing the host plant’s own disease resistance. Jin et al. (2024) revealed that in cotton plants treated with HB3S-20, the activities of SOD and POD enzymes increased over time and peaked at 96 h post-treatment. SOD activity rose from 275 to 575 U/mg protein, and POD activity increased from 85 to 225 U/mg protein. These results confirm that strain HB3S-20 induces disease resistance in cotton, thereby enhancing its defense against pathogens (Figure 4 and Table 4).

**Figure 4:**
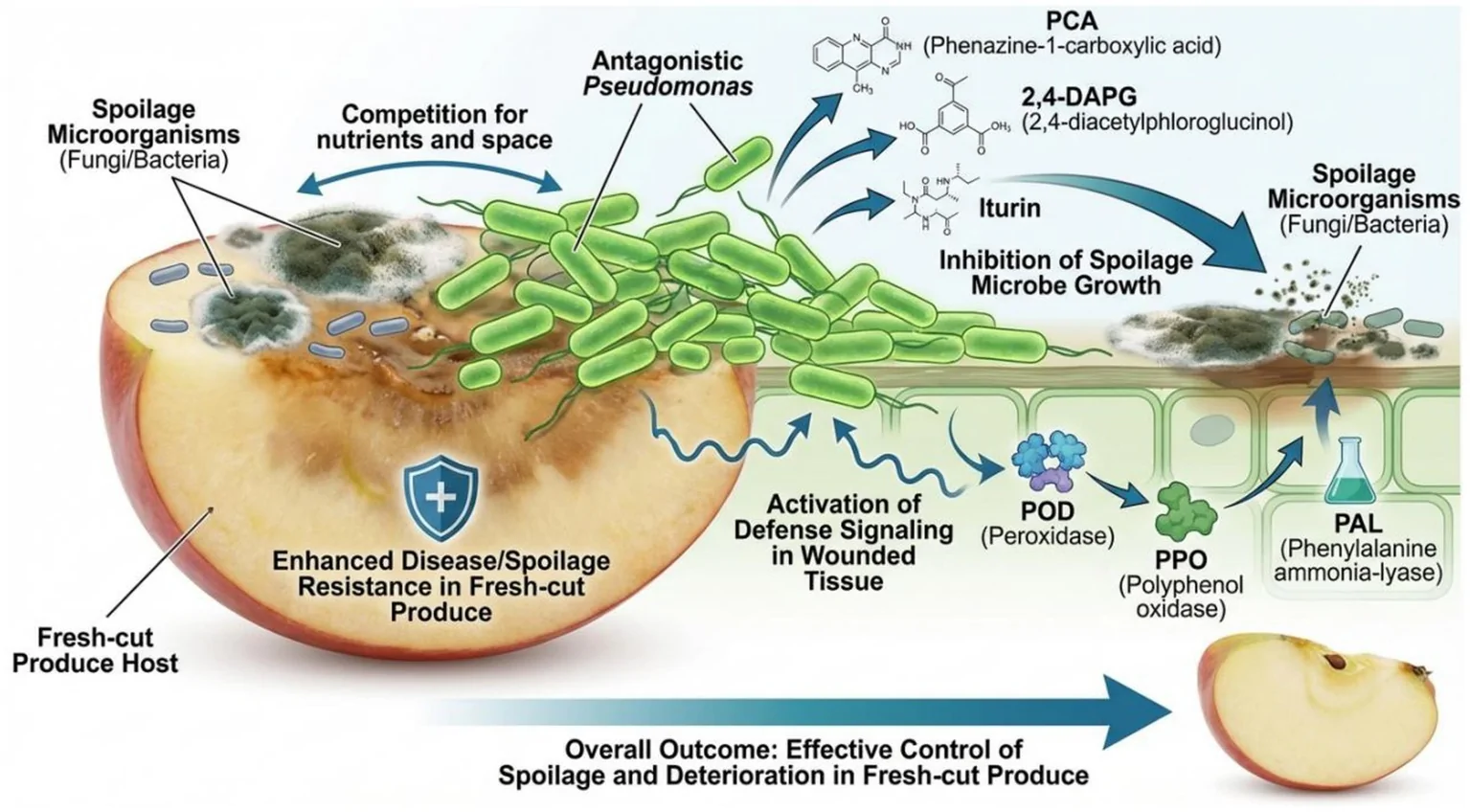
Mechanism of action of antagonistic *Pseudomonas*.

**Table 4:** Representative *Pseudomonas* with effective biocontrol efficacy and their modes of action.

Source	Types	Disease name	Types of fruits and vegetables	Mechanism of action	References
Found widely in soil, water bodies, and especially the plant rhizosphere.	*Pseudomonas fluorescens*	Gummy stem blight	Muskmelon	*Pseudomonas fluorescens*m18 controls disease primarily by producing the antimicrobial metabolite phenazine-1-carboxylic acid (PCA) and inducing systemic resistance in muskmelon.	Zhu et al. (2001)
		Fusarium wilt	Cucumber	*Pseudomonas fluorescens* achieves biocontrol by directly inhibiting or killing pathogens through the production of various antimicrobial substances such as DAPG, PCA, and pyrrolnitrin.	Yue et al. (2008)
		Damping-off	Sugar beet	By rapidly proliferating and activating beneficial rhizosphere communities to achieve dominance, it also triggers the sugar beet’s defense system, thereby elevating disease resistance.	Liu and Xian (1995)
		Blue mold	Apple	The production of diffusible antimicrobial metabolites, including antibiotics like 2,4-diacetylphloroglucinol, directly inhibits *Penicillium expansum* and *P. niveum*, the causal agents of apple blue mold.	Etebarian et al. (2005)
		Blue mold	Citrus	It effectively controls blue mold caused by *Penicillium italicum* in Citrus by rapidly competing for nutrients and space at wound sites, and by inducing fruit chitinase and β-1,3-glucanase activity to enhance systemic resistance.	Wang et al. (2018)
		Gray mold	Grape	It effectively controls postharvest gray mold in grapes by producing volatile organic compounds (VOCs) such as aldehydes and ketones to directly inhibit *Botrytis cinerea* spore germination and mycelial growth, rapidly competing for space and nutrients on the fruit surface, and disrupting pathogen hyphae through mycoparasitism.	Wei et al. (2021)
From rhizosphere soil, internal plant tissues, and natural water bodies.	*Pseudomonas protegens*	Black rot	Cabbage	It effectively suppresses pathogens through a combination of mechanisms: competition for nutrients and space, production of antimicrobials such as pyoluteorin and DAPG, and induction of plant systemic resistance.	Wang (2021)
		Bacterial fruit blotch	Muskmelon	It achieves synergistic disease control by producing multiple antimicrobials (e.g., DAPG, pyoluteorin, pyrrolnitrin, 2-hydroxyphenazine), possessing capabilities like phosphate solubilization, protease secretion, biofilm formation, and HCN production, alongside competition for nutrients/space and induction of plant resistance.	Zhao L. N. et al. (2024) and Zhao Z. X. et al. (2024)
		Gray mold	Tomato	It directly inhibits spore germination and mycelial growth of pathogenic fungi by producing various antimicrobial substances, including pyrrolnitrin, DAPG, pyoluteorin, siderophores, HCN, and proteases.	Chang et al. (2011)
Widely distributed in natural environments such as soil, water bodies, and plant rhizospheres.	*Pseudomonas aeruginosa*	Fusarium wilt	Tomato	By producing a macromolecular proteinaceous antimicrobial substance, it effectively disrupts the hyphal structure of pathogenic fungi and inhibits their growth, while also exhibiting a growth-promoting effect on tomato seedlings.	Hao et al. (2011)
		Bacterial canker、Bacterial spot	Tomato	By producing antimicrobial substances such as siderophores, HCN, and proteases, and competing for rhizosphere nutrients and colonization sites, it significantly inhibits pathogen growth.	Ghadamgahi et al. (2022)
		Late blight	Potato	It strongly inhibits mycelial growth and spore germination by secreting volatile and non-volatile metabolites such as phenazines and HCN.	Ghadamgahi et al. (2022)
		Taro leafmold	Taro	By producing metabolites such as lipase and siderophores, it effectively inhibits the infection and spread of pathogens.	Ghadamgahi et al. (2022)
		Fusarium Wilt	Banana	It effectively suppresses pathogens by producing antimicrobials such as siderophores, HCN, proteases, chitinase, and β-1,3-glucanase, along with various secondary metabolites, and extensively colonizes root, stem, and leaf tissues.	Singh et al. (2023)
Mainly sourced from plant surfaces (e.g., leaves, fruits) and their surrounding environments like soil and water.	*Pseudomonas syringae*	Apple ring rot	Apple	By producing antimicrobial metabolites such as lipopeptides and siderophores, it significantly inhibits pathogenic fungi including *Botryosphaeria* spp., achieving a control efficacy of over 70% against both fruit and branch ring rot.	Qingdao Agricultural University (2020)
		Gray mold	Tomato	*Pseudomonas syringae* ZY-1 directly inhibits *Botrytis cinerea*, the tomato gray mold pathogen, through antimicrobial metabolites in its fermentation broth, demonstrating strong control efficacy against the disease.	Northeast Agricultural University (2014)

### 2.5 Preservation of fresh-cut fruits and vegetables by antagonistic *Trichoderma*

Antagonistic *Trichoderma* are primarily isolated from soil, rhizosphere soil, and decaying organic matter (Ma et al., 2025b). Common species such as *Trichoderma harzianum*, *T. viride*, and *T. asperellum* are applied in preserving fruits and vegetables including melon, banana, tomato, and strawberry. Their mechanisms against postharvest diseases mainly include competition for nutrients and space, mycoparasitism, antibiosis, and induction of plant resistance (Yang and Yang, 2021).

In terms of competition for nutrients and space and mycoparasitism, Huo et al. (2022) demonstrated that on dual-culture plates, the *Trichoderma viride* strain Tv-1511 occupied three-fourths of the area by invading, covering, or surrounding colonies of *Fusarium oxysporum, F. proliferatum, F. moniliforme, and Botrytis cinerea*, with respective inhibition rates of 68.50, 66.65, 68.96, and 59.81%. This visual evidence indicates competitive suppression via rapid growth and nutrient/space competition. Furthermore, microscopic observation revealed that Tv-1511 hyphae attached to and coiled around the hyphae of *F. oxysporum, F. moniliforme, and B. cinerea*, causing hyphal deformation, fracture, and lysis, thereby confirming the mycoparasitism mechanism.

In addition to competition and mycoparasitism, antibiosis is also an important way for *Trichoderma* to exert its biocontrol effect. Chen et al. (2013) identified a *Trichoderma harzianum* strain that produces both non-volatile (41.33% inhibition) and volatile (11.49% inhibition) antibiotics, forming a three-dimensional antimicrobial network. Bioactive secondary metabolites constituted 30.28% of the total extract, confirming an antibiosis mechanism.

Finally, *Trichoderma* can also enhance its biocontrol efficacy by inducing the plant’s own resistance. Zhang et al. (2025) reported that all *Trichoderma* strains effectively controlled Fusarium wilt of watermelon, with efficacy ranging from 33.33 to 71.67%. For the most effective strain, XHMG-3, plant height increased from 14.97 to 16.80 cm and leaf area from 26.01 to 33.12 cm^2^, confirming induced plant resistance as the control mechanism (Figure 5 and Table 5).

**Figure 5:**
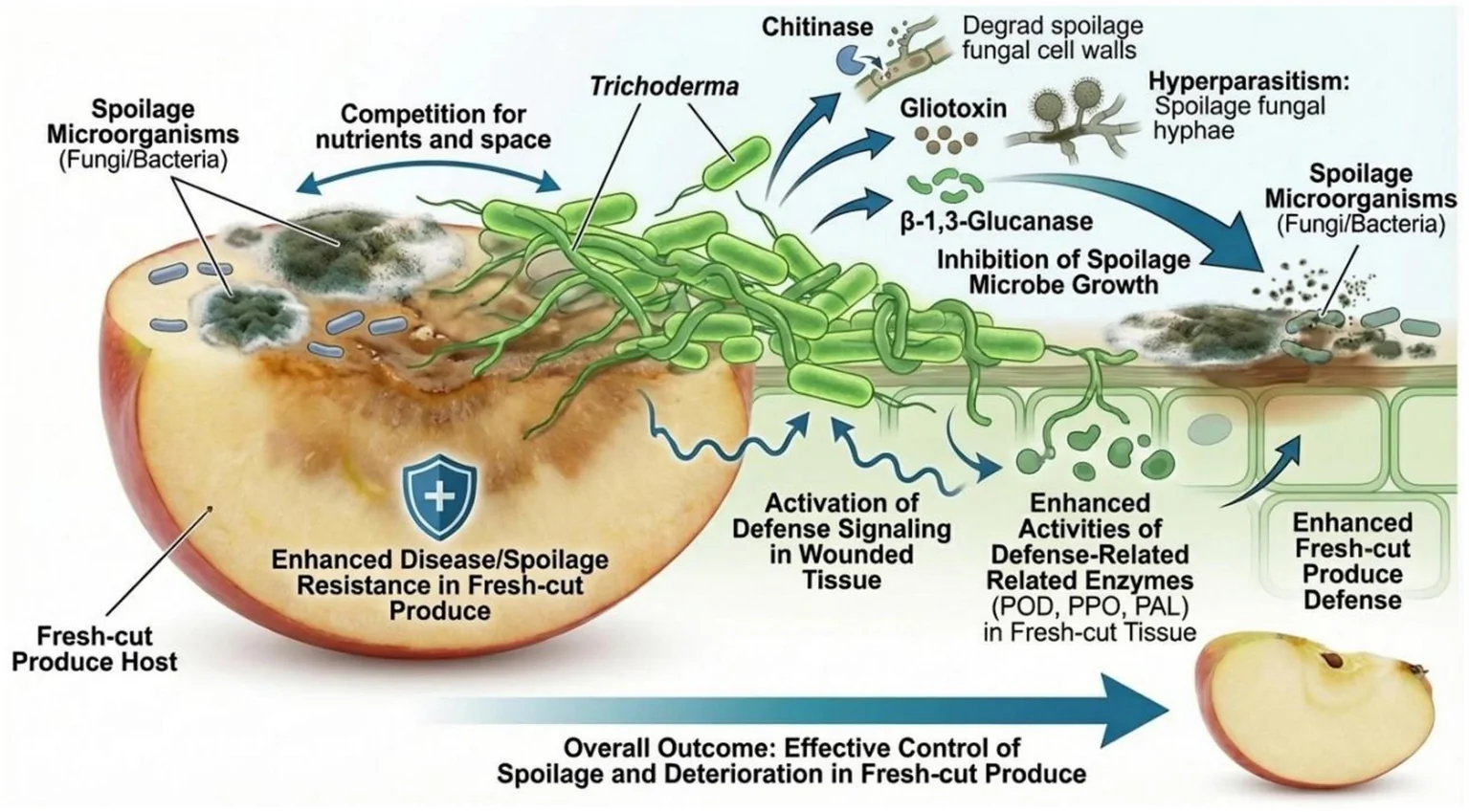
Mechanism of action of antagonistic *Trichoderma*.

**Table 5:** Representative *Trichoderma* with effective biocontrol efficacy and their modes of action.

Source	Types	Disease name	Types of fruits and vegetables	Mechanism of action	References
From natural environments: soil, the rhizosphere, and decomposing plant matter.	*Trichoderma harzianum*	Early blight	Tomato	*Trichoderma harzianum* M408 achieves biocontrol of tomato blight by competing for nutrients and space to effectively inhibit the growth of *Alternaria solani* W5.	Su et al. (2025)
		Fusarium wilt	Bitter melon	*Trichoderma harzianum* FJAT-9040 achieves biocontrol against Fusarium wilt of bitter melon by inhibiting the pathogen through competition, mycoparasitism, and antibiosis, along with the production of antimicrobials such as trichodermin, gliotoxin, and cell wall-degrading enzymes.	Xiao et al. (2015)
		Fusarium wilt	Muskmelon	*Trichoderma harzianum* DQ002 controls Fusarium wilt of muskmelon by directly inhibiting pathogen mycelial growth with antimicrobials from its fermentation broth and inducing systemic resistance via activation of plant PAL and β-1,3-glucanase defense systems.	Li et al. (2023)
		Fusarium wilt	Chili	*Trichoderma harzianum* T21 achieves biocontrol by inhibiting the growth and spore germination of Fusarium wilt pathogen in pepper via competition, mycoparasitism, and antimicrobial substances (e.g., chitinase, β-1,3-glucanase) from its fermentation broth.	Liang et al. (2022)
		Southern corn leaf blight	Corn	*Trichoderma harzianum* SH2303 primarily activates systemic resistance in maize, significantly enhancing the activity and gene expression of defense enzymes (PAL, SOD), thereby inducing durable resistance against Southern corn leaf blight.	Ma et al. (2014)
		Strawberry root rot	Strawberry	*Trichoderma harzianum* NBL-Z1 controls strawberry root rot via effective rhizosphere colonization and direct inhibition of pathogens (e.g.*, F. oxysporum*) through secreted antimicrobials, including antibiotics and cell wall-degrading enzymes.	Qi et al. (2021)
From cellulose-rich decomposing plant material and soil.	*Trichoderma viride*	Fusarium wilt	Watermelon	*Trichoderma viride* inhibits pathogens primarily through competition for nutrients and space, as well as mycoparasitism. It grows rapidly on plates, covering and invading pathogen colonies, leading to hyphal breakage and distortion.	Zhang et al. (2010)
		Fusarium wilt	Banana	*Trichoderma viride* inhibits Fusarium wilt of banana through competition for nutrients and space, as well as mycoparasitism.	Zhang et al. (2010)
		Damping-off	Muskmelon	*Trichoderma viride* Tv286 promotes muskmelon growth and induces resistance to damping-off by systemically enhancing physiological functions, including chlorophyll content, root activity, key enzyme activities, and nutrient levels.	Lian et al. (2024)
		Fusarium wilt	Banana	*Trichoderma viride* H6 induces systemic resistance in banana, significantly elevating the activities of defense enzymes (POD, PAL, CAT, SOD, PPO) and thereby enhancing plant immunity to Fusarium wilt.	Xin et al. (2013)
		Gray mold	Tomato	*Trichoderma viride* T25 controls tomato gray mold primarily through competition for nutrients and space, mycoparasitism, and induction of plant systemic resistance, producing antimicrobials that include volatile metabolites and secretions.	Jin (2007)
		Fusarium wilt	Cucumber	*Trichoderma viride* T23 controls Fusarium wilt of cucumber primarily by inducing plant systemic resistance, as manifested by the activation of defense enzymes (PAL, POD, PPO, CAT) and promotion of lignin and phytoalexin synthesis.	Zhuang et al. (2005)
From soil, decomposing plant material, and specific agricultural environments.	*Trichoderma asperellum*	Anthracnose	Strawberry	*Trichoderma asperellum* GYSW-6 m1 controls strawberry anthracnose through synergistic mechanisms including competition for nutrients and space, mycoparasitism, and production of non-volatile antimicrobial metabolites.	Zhao et al. (2020)
		Fusarium Wilt	Tomato	The applied mechanisms primarily include competition for nutrients and space, mycoparasitism, antibiosis, and induction of plant resistance. The produced antimicrobial substances comprise volatile organic compounds (VOCs), cell wall-degrading enzymes (e.g., chitinase, β-glucanase, cellulase, protease), and antibiotic compounds such as pyrones, terpenoids, steroids, and polyphenols.	Lu (2024)
		Fusarium Wilt	Bitter melon	*Trichoderma asperellum* exerts biocontrol by directly inhibiting pathogen growth through the production of various antimicrobials, such as chitinase, β-1,3-glucanase, and antimicrobial peptides.	Du (2019)
		Fusarium Wilt	Banana	*Trichoderma asperellum* PZ6 enhances banana immunity against Fusarium wilt by activating defense enzyme systems (e.g., SOD, CAT) and inducing systemic resistance.	Qin et al. (2017)
		Citrus black spot	Citrus	The mechanisms of *Trichoderma asperellum* against citrus black spot (CBS) primarily include mycoparasitism and antibiosis, with the production of key antimicrobials such as laccase and other extracellular degrading enzymes.	Liu (2021)

### 2.6 Safety assessment of antagonistic microorganisms for fresh-cut produce preservation

The application of live antagonistic microorganisms in fresh-cut produce preservation requires systematic safety assessment based on the Generally Recognized As Safe (GRAS) principle. Since such products are typically consumed raw without heat treatment, the selected microorganisms must be non-pathogenic, devoid of virulence factors, and free of transferable antibiotic resistance genes. Lactic acid bacteria, such as *Lactobacillus plantarum* and *Lactobacillus pentosus*, have a long history of safe use in traditional fermented foods and are therefore preferred candidates for preservation applications. Genomic studies have confirmed that *L. plantarum* BX62 effectively extends the shelf life of fresh-cut apples while significantly inhibiting spoilage microorganisms, with genome-wide analysis revealing no virulence or resistance genes (Zhao et al., 2021). Similarly, *L. pentosus* MS031, isolated from Sichuan pickles, produces antimicrobial substances that specifically inhibit foodborne pathogens without cytotoxicity to human cells, as demonstrated through combined metabolomic and genomic analyses (Yi et al., 2020).

Safety evaluation must cover the entire production chain from farm to table. Pre-harvest spraying of lactic acid bacteria on leafy vegetables such as lettuce and spinach can reduce the abundance of high-risk pathogens, including *Listeria monocytogenes* and *Escherichia coli* O157: H7, thereby lowering the microbiological safety risks of ready-to-eat products (Yin et al., 2023). Beyond lactic acid bacteria, most of the antagonistic microorganisms introduced in this chapter—including *Bacillus* spp., *Trichoderma* spp., antagonistic yeasts, and epiphytic *Pseudomonas* strains—meet GRAS safety standards. These strains are mostly isolated from plant surfaces and are naturally colonized on fruits and vegetables, posing no health threat to consumers under normal consumption conditions. Encapsulating live cells in edible composite film systems can stabilize strain activity and reduce excessive attachment of viable cells on produce surfaces, simultaneously ensuring preservation efficacy and application safety (Temiz and Özdemir, 2021).

Compared with chemical preservatives, the metabolites secreted by antagonistic microorganisms, such as lactic acid and bacteriocins, are naturally occurring food components and carry no risk of chemical residues. Low-temperature cold chain storage further optimizes safety control: it suppresses the overgrowth of antagonistic strains and limits the synthesis of harmful secondary metabolites in some *Bacillus* species; meanwhile, the cold chain enhances the inhibitory efficacy of antagonists, thereby reducing the required dosage and further minimizing microbial residue risks on fresh-cut produce surfaces. In conclusion, when combined with standardized strain identification, toxicological testing, and full-chain cold chain management, antagonistic microorganisms demonstrate sufficient safety and favorable application prospects for fresh-cut produce preservation.

## 3 Synergistic mechanisms of cold chain logistics and antagonistic microorganisms

The combination of low-temperature cold chain storage and antagonistic microorganisms is not a simple superposition of two independent preservation technologies, but a synergistic strategy with overall efficacy exceeding the sum of individual effects. The synergistic preservation effect is embodied in three core dimensions: the cold chain environment creates favorable conditions for the targeted functioning of antagonistic microorganisms, antagonistic microorganisms compensate for the inherent antibacterial deficiency of low-temperature storage, and the combined strategy comprehensively improves the stability and safety of the preservation system.

### 3.1 Cold chain conditions facilitate antagonistic microorganism colonization and activity

Low-temperature conditions serve as a critical foundation for improving the colonization efficiency and functional stability of antagonistic microorganisms, which constitutes an important basis for achieving synergistic preservation.

#### 3.1.1 Cold-induced inhibition of the competing microbiota

Although low temperature cannot completely eliminate all spoilage microorganisms, it significantly inhibits the rapid proliferation of most mesophilic pathogens and spoilage bacteria (General Office of the State Council, 2021). This effect reduces the competitive pressure that exogenously applied antagonists face for nutrients and spatial niches on fresh-cut produce surfaces, allowing them to colonize wound tissue more efficiently and rapidly establish a dominant population.

#### 3.1.2 Maintenance of host tissue integrity via cold storage

Cold storage reduces the respiration rate and ethylene biosynthesis of fresh-cut fruits and vegetables, delays tissue senescence, and maintains cell integrity and tissue firmness (Wang et al., 2025a). Healthy and stable host tissues provide a more stable colonization substrate for antagonistic microorganisms and reduce the risk of pathogen invasion via mechanically damaged tissues, which indirectly strengthens the biocontrol effect.

#### 3.1.3 Enhancement of antimicrobial metabolite stability via controlled cold storage

A variety of antagonistic microorganisms and their active metabolites exhibit higher structural stability under low-temperature conditions. For instance, nisin, a typical bacteriocin derived from *Bacillus* spp., can insert into the cell membrane of Gram-positive bacteria more stably and form pores under cold chain conditions, showing enhanced membrane penetration and improved stability (Cao et al., 2023); psychrotolerant strains such as antagonistic lactic acid bacteria can maintain long-term metabolic activity throughout cold chain storage and distribution (Du et al., 2025).

### 3.2 Antagonistic microorganisms compensate for inherent limitations of cold chain

The core limitation of single cold chain preservation lies in its failure to effectively inhibit psychrotrophic pathogens, which are the dominant factors causing quality deterioration and safety risks in fresh-cut produce (Chen et al., 2012). Antagonistic microorganisms precisely fill this functional gap.

#### 3.2.1 Targeted inhibition of psychrotrophic pathogens via antagonistic strains in cold chain

Specific antagonistic strains exert significant inhibitory effects on key psychrotrophic pathogens that proliferate under refrigeration, including *Listeria monocytogenes*, spoilage *Pseudomonas* spp., and *Clostridium botulinum*. For example, lactic acid bacteria and *Bacillus* strains can produce bacteriocins such as nisin that maintain stable bactericidal activity at low temperatures and directly inhibit or kill these cold-adapted pathogens (Charest et al., 2024).

#### 3.2.2 Multi-target cooperative control of psychrotrophs via multi-modal antagonism in cold chain

Unlike low temperature, which merely slows microbial metabolism, antagonistic microorganisms exert antibacterial effects via multiple concurrent mechanisms, including competition for nutrients and space, secretion of antibacterial metabolites, mycoparasitism, and induction of host resistance. This multi-modal action reduces the likelihood of resistance development and ensures more reliable preservation efficacy against cold-adapted pathogens (Li et al., 2012).

Combining cold chain logistics with antagonistic microorganisms establishes a dual preservation framework that integrates low-temperature physiological regulation with targeted biological inhibition. The cold chain enhances colonization and functional stability of exogenous antagonists, while they compensate for the insufficient suppression of psychrotrophic spoilage bacteria and pathogens by cold storage alone. This synergistic strategy outperforms either treatment alone in inhibiting spoilage microbiota, retaining quality, and ensuring microbial safety, providing a theoretical basis for green preservation of fresh-cut produce.

## 4 Future trends of antagonistic microbes in fruit and vegetable preservation

### 4.1 Technological advances in precise screening of antagonistic microbes

The screening of antagonistic microbes is transitioning from traditional broad-spectrum methods to targeted, functionally defined precision screening. This shift is driven by the integration of multi-omics and molecular biology tools, particularly to meet the screening demands of cold chain fresh-cut preservation for cold-adapted antagonistic strains. Unlike inefficient traditional methods that rely on phenotypic assays (e.g., antagonistic zone assay) and lack specificity, modern precision screening enables targeted mining of functional strains adapted to low-temperature environments through analysis of pathogen virulence mechanisms and antagonistic microbial networks under refrigeration conditions.

In controlling banana Fusarium wilt, researchers targeted and identified hub strains such as *Pseudomonas* sp. OTU26 and *Bacillus velezensis* OTU118 from a pepper-banana rotation system. This was achieved by *in situ* tracking of soil microbial community assembly patterns, combined with high-throughput sequencing, co-occurrence network analysis, and pure culture techniques. The fermentation broth and volatile compounds of these strains still maintain stable antifungal activity under conventional refrigeration temperatures of cold chain circulation. In pot experiments, the disease incidence of banana *Fusarium* wilt was reduced from 53 to 31%, demonstrating the notable advantage of targeted screening for strains applicable to low-temperature preservation (Hong et al., 2023). Wang et al. (2024) systematically analyzed the basis for antimicrobial substance biosynthesis in the strain by integrating genomics, metabolomics, and other technologies. This strategy not only genetically verified the presence of known antibiotics but also predicted potential novel antimicrobials with stable activity at low temperatures, enabling the correlation of antimicrobial phenotypes with their underlying genetic mechanisms and chemical compositions, which lays a molecular foundation for the application of strains in long-distance cold chain transportation.

In addition, omics technologies, such as transcriptomics and proteomics (Cao et al., 2013), can be used to analyze the antimicrobial protein networks of the strains under cold stress. Researchers can clarify the cold-responsive expression rules of antimicrobial functional proteins, which provides molecular-level support for identifying strong, safe, and cold-tolerant antagonistic microbes suitable for cold chain scenarios. Future screening will also employ novel gene-editing methods, such as CRISPR-Cas9 (Liu et al., 2021), to perform targeted genetic modifications for cold adaptation. Gene editing is used to upregulate the expression of cold-resistance genes and low-temperature antibacterial genes of antagonistic strains, rather than blindly enhancing general antibacterial ability. The modified strains can maintain high antagonistic activity and metabolic stability throughout the entire cold chain storage and transportation cycle, and produce more stable antagonistic functional metabolites under refrigeration conditions.

### 4.2 Optimization of antagonistic microbe release characteristics integrated with cold chain shelf-life prediction

Enhancing the release stability and duration of antagonistic microbes and their antibacterial metabolites on produce surfaces under low-temperature conditions is a core challenge for cold chain preservation. Optimizing sustained-release formulations and integrating intelligent shelf-life prediction models tailored to cold chain environments are critical to overcoming the instability of biocontrol agents during long-term low-temperature storage and transportation. Unlike traditional microbial formulations that are prone to rapid inactivation and uneven release under variable cold chain temperatures, novel sustained-release carrier systems enable stable, slow, and controlled release of antagonistic substances throughout the entire cold chain distribution process (Cao, 2015). For instance, iturin A/chitosan nanocapsules (average size: 553.4 nm) with high thermal and temperature-shock stability were successfully prepared to adapt to cold chain logistics fluctuations. *In vitro* tests demonstrated their excellent sustained-release property under low-temperature conditions, achieving a 70.61% cumulative release of iturin A by day 16. When applied to Kyoho grapes and stored under simulated cold chain conditions, these nanocapsules steadily maintained antibacterial activity, reduced decay incidence by 52.28% and weight loss by 41.2%, and effectively extended the cold-chain shelf life beyond 16 days (Li et al., 2024a; Li et al., 2024b).

Multiple physiological and microbial indicators, including respiratory intensity, weight loss rate, decay incidence, and TSS/TA variation, can serve as core input parameters for cold chain shelf-life prediction models. By further integrating the low-temperature sustained-release characteristics of IA/CS nanocapsules, real-time electronic nose data (O₂, CO₂, and ethylene accumulation in cold storage), hyperspectral image information (400–1,000 nm), and routine laboratory indices (firmness, soluble solids, and microbial load), high-precision hybrid models such as Random Forest and CNN-LSTM can be constructed. Incorporating a dynamic weight adjustment mechanism that adapts to low-temperature storage, transportation, and retail shelf stages enables the establishment of an intelligent and dynamic prediction system specifically applicable to the cold chain preservation efficacy of antagonistic microorganisms (Senge et al., 2025).

Chen (2022) developed a cyclodextrin-based metal–organic framework (N, S-CD/CD-MOF) sustained-release carrier, which achieves continuous release of active antibacterial components over 12–15 days and provides long-term quality protection for fresh-cut cantaloupe during cold storage. Such porous carrier systems possess excellent low-temperature tolerance and are highly compatible with antagonistic microbial cells, enabling stable immobilization and temperature-responsive controlled release of biocontrol strains under cold chain conditions. CD-MOFs and similar functional materials exhibit stable loading capacity and environment-responsive release behavior under refrigeration fluctuations (Alotaibi et al., 2023), providing a reliable technical foundation for improving the low-temperature adaptability and long-term preservation stability of antagonistic microorganisms in industrial cold chain circulation.

### 4.3 Intelligent optimization of antagonistic microbes through multi-technology integration for cold chain adaptability

The combined application of antagonistic microorganisms with natural polysaccharides, cell-wall-degrading enzymes, and plant essential oils can produce significant low-temperature synergistic antibacterial effects, which is crucial for stabilizing the biocontrol performance of microbial agents during whole-process cold chain preservation. Single antagonistic strains often exhibit decreased metabolic activity and insufficient colonization ability under low-temperature stress; composite modification can effectively compensate for the functional weaknesses of single microbial agents in cold environments and further enhance their cold-stable antibacterial activity. A previous study confirmed that a composite film embedded with iturin A-producing antagonistic *Bacillus* and chitosan reduced mango anthracnose incidence by 62.3% under low-temperature storage. Synergistic analysis showed an additive interaction coefficient (AICE) greater than 1.5 for the combined treatment. This composite system also lowered the mango respiration peak by 50.5% and activated host antioxidant and defense enzymes under cold chain conditions, achieving dual cold-chain preservation benefits through microbial inhibition and host cold-resistance enhancement (Li et al., 2024a; Li et al., 2024b). Additional studies have confirmed that the combination of cell wall-degrading enzymes secreted by cold-tolerant *Trichoderma* and lipopeptide antibiotics produced by epiphytic *Pseudomonas* exhibits significantly stronger synergistic inhibition against postharvest pathogenic fungi under refrigeration conditions compared with single components (Woo et al., 2002). Research by Arrebola et al. (2010) demonstrated that the combined application of *Bacillus amyloliquefaciens* PPCB004 and citronella essential oil maintains stable synergistic antibacterial activity at low temperature. The strain continuously produces cold-stable antimicrobial lipopeptides (iturin A, fengycin, surfactin) and volatile acetoin during cold chain storage, while citral from the essential oil assists in destroying fungal cell membranes under low-temperature static storage conditions. The combined treatment achieved inhibition rates of 50, 42, and 82% against postharvest gray mold, blue mold, and rhizopus rot, respectively, in cold-stored peach fruit.

With the rapid development of cold chain intelligent preservation, artificial intelligence (AI) and machine learning technologies have gradually become efficient auxiliary tools for the directional optimization of cold-chain-adapted antagonistic microbial composite systems. Traditional screening of composite preservation systems relies on a large number of low-temperature experiments and has low efficiency, whereas AI technology can accurately predict the low-temperature compatibility, cold-stable antibacterial activity, and synergistic mechanisms of antagonistic microbes and auxiliary substances. For key browning and spoilage targets such as polyphenol oxidase (PPO) and tyrosinase that remain active under refrigeration, AI can predict the low-temperature binding affinity of natural antibacterial and antioxidant compounds (Cherneva et al., 2025), rapidly screen composite formulations suitable for cold chain environments, and avoid formulation failure caused by low-temperature inactivation. Meanwhile, deep learning generative models such as generative adversarial networks (GANs) can directionally design novel low-temperature-resistant natural preservative molecules that match the metabolic characteristics of antagonistic microbes (Zhou H. J. et al., 2025; Zhou M. Y. et al., 2025), forming highly compatible composite cold-chain preservation systems.

In future cold chain preservation research, multi-factor intelligent optimization models based on machine learning can be constructed to accurately screen the optimal compound ratios and application parameters of antagonistic microorganisms and natural additives specifically for long-term low-temperature storage. This integrated intelligent approach can achieve precise regulation of microbial synergistic effects under cold chain conditions, further improve the stability and controllability of biological preservation systems in industrial cold chain circulation, and provide new technical support for the intelligent and green upgrading of fresh-cut fruit and vegetable cold chain preservation.

### 4.4 Construction of a whole-chain safety and quality traceability system

A whole-chain safety and quality traceability system for production, storage, transport, and sales is key to making sure the safety and quality of fruit and vegetable preservation are stable and to helping the industry move from passive prevention to active traceability. This system uses digital tools to remove information barriers at all stages, allowing clear supervision from farm to table and addressing the long-standing limitation of prioritizing prevention over traceability.

Blockchain technology serves as the core foundation of the traceability system. And it uses its features like decentralization and data immutability, so it provides robust technical support for data authenticity and reliability. The system uses a mixed storage model with on-chain and off-chain storage. Critical data is stored on the blockchain, and key parameters like temperature, humidity, and gas are collected by IoT devices, and this makes data collection and traceability efficient and reliable (Morais et al., 2024). One study built a visual traceability framework based on consortium blockchain. It uses a risk assessment model and visualization tools, which helps regulators find high-risk areas and time periods for preservation problems at the macro level and trace the flow and causes of bad batches at the micro level. This provides a new technological paradigm for improving supply chain transparency and reliability (Hao et al., 2020).

Shi Liang (Shi, 2020) developed a traceability system that uses blockchain and IoT for fruits and vegetables. This system uses blockchain’s safe and shared storage, so it ensures that the environmental data (such as temperature, humidity, and gas) from IoT devices are true. It also uses Logistic and HMM models, which allows it to trace and check the storage conditions throughout the entire process, greatly improving the transparency of cold chain and storage management. The mixing of different technologies is now a key direction for quality testing. One study used a CNN to obtain the color, texture, and shape from fruit images, and used a BiLSTM to obtain time links between features, as well as optimization methods. This combined model achieved a high accuracy of 97.76% in freshness testing (Yuan et al., 2024a). NIRS finds the vibration signals of chemical bonds like C-H and H-O, so it can quickly and safely measure quality indicators like firmness, sugar content, and vitamin C (Guo et al., 2024). The combination of HSI and DL provides a feasible way to quickly, accurately, and safely check fruit and vegetable quality and safety, and it helps with smart grading and traceability in the industry (Yang et al., 2025). In the future, the whole-chain traceability system will be built on deeper cooperation between different technologies. It will move towards predictive tracing, standardized teamwork, and full tracking. This will shift from just looking back at problems to predicting and controlling them early, and it will give key support for the industry’s high-quality growth.

## 5 Conclusion

Cold chain logistics provide a basic storage environment for fresh-cut fruits and vegetables, but their inhibitory effect on cold-tolerant pathogens is limited. Therefore, incorporating antagonistic microorganisms enables a synergistic preservation strategy combining “low-temperature suppression and biological antagonism.” This approach employs multiple mechanisms, such as competition for nutrients and space, production of antimicrobial metabolites, induction of host resistance, and parasitism, to precisely target pathogens that remain active under low temperatures. This significantly delays decay and preserves the quality of produce. Future research should prioritize targeted screening of antagonistic microorganisms, development of intelligent controlled-release carriers, integration of multiple technologies, and establishment of a full-chain traceability system. These efforts will promote the development of this strategy toward greater efficiency, intelligence, and industrial applicability.
